# Big number, big body: Jersey numbers alter body size perception

**DOI:** 10.1371/journal.pone.0287474

**Published:** 2023-09-07

**Authors:** Leon T. Shams, Alisha Föry, Achint Sharma, Ladan Shams

**Affiliations:** 1 Department of Psychology, University of California, Los Angeles, Los Angeles, California, United States of America; 2 Department of Bioengineering, University of California, Los Angeles, Los Angeles, California, United States of America; 3 Neuroscience Interdepartmental Program, University of California, Los Angeles, Los Angeles, California, United States of America; Universita degli Studi di Torino, ITALY

## Abstract

Vision has been shown to be an active process that can be shaped by top-down influences. Here, we add to this area of research by showing a surprising example of how visual perception can be affected by cognition (i.e., cognitive penetration). Observers were presented, on each trial, with a picture of a computer-generated football player and asked to rate the slenderness of the player on an analog scale. The results of two experiments showed that observers perceived athletes wearing small jersey numbers as more slender than those with high numbers. This finding suggests that the cognition of numbers quantitatively alters body size perception. We conjecture that this effect is the result of previously learned associations (i.e., prior expectations) affecting perceptual inference. Such associations are likely the result of implicit learning of the statistical regularities of number and size attributes co-occurrences by the nervous system. We discuss how these results are consistent with previous research on statistical learning and how they fit into the Bayesian framework of perception. The current finding supports the notion of top-down influences of cognition on perception.

## Introduction

**Fig 1:**
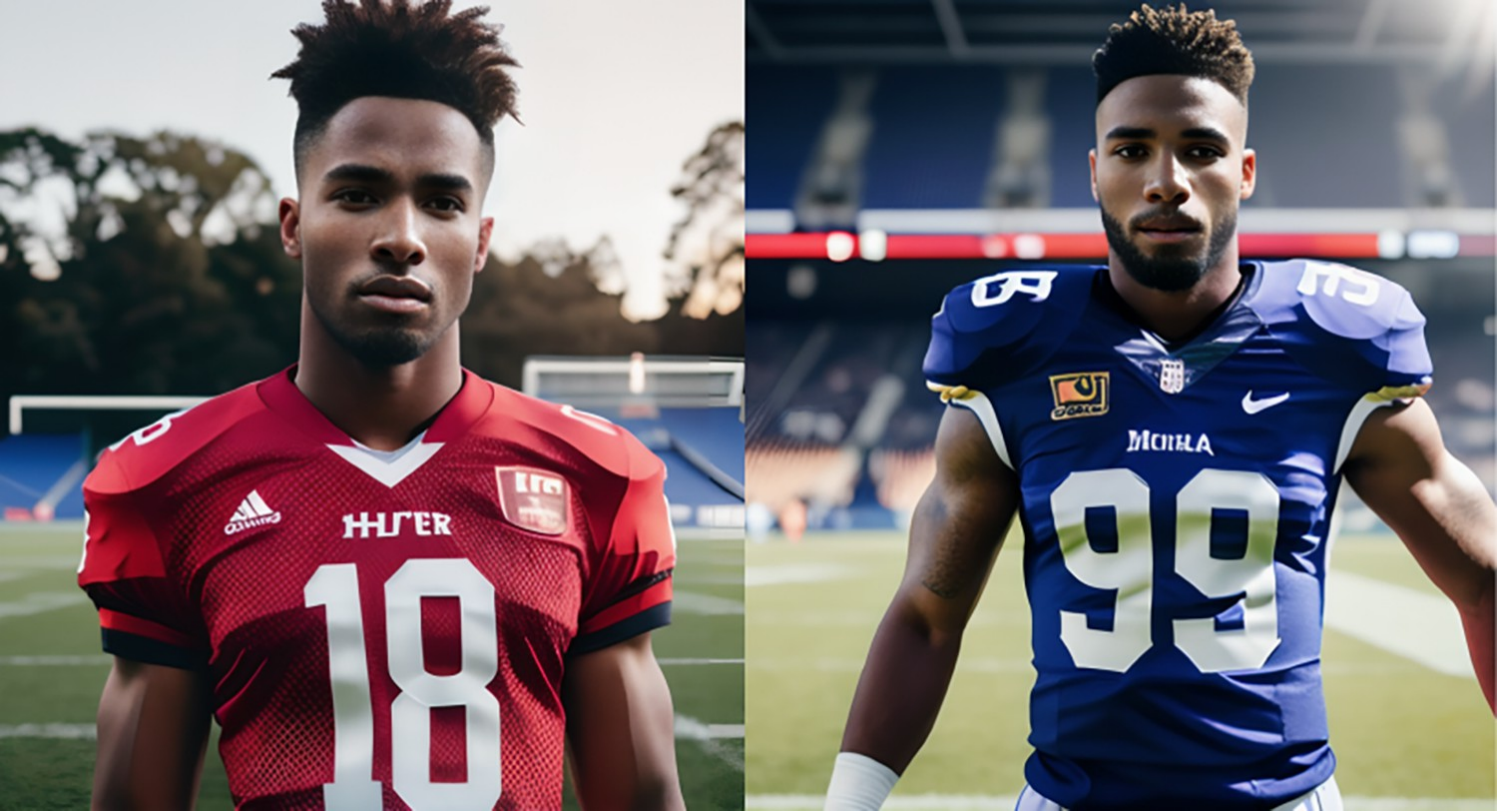
Two football players, one (left) with a low jersey number, and one (right) with a large jersey number.

When athletes choose jersey numbers (see Fig 1), they tend to go for low instead of high numbers as reported in a survey performed by ESPN [1]. Football players argue that small numbers make them look faster, more slender, and more agile. Given the fact that the way we perceive the world is considerably influenced by our prior knowledge (for an overview see e.g., [2–5]) there may be more behind these observations than simple, random preferences of the athletes. In our daily life, numbers written on objects usually represent the magnitude of the objects such as the numbers written on a bag of sugar or rice in the supermarket, or labeled weights in the gym. The higher the number the bigger, or more massive the represented object generally is. Such statistical regularities may be stored in the brain and shape future perception. Previous research has reported various examples of cognitive penetration and top-down influences on sensory processing (see [6–8] for overviews). Perhaps one of the most compelling demonstrations of the effect of cognition on perception is the radical change in the perception of sine-wave speech stimuli induced by cognitive knowledge (e.g., [9, 10]). Sine-wave speech stimuli are impoverished speech stimuli that are generally perceived as squeaky noise, however, once the observer is told that what they are hearing is speech, the phenomenology is suddenly changed into a specific speech utterance (the specific utterance that was corrupted by noise), and the observer can no longer go back to hearing the squeaky noise that they heard before. The top-down influence of cognition on perception is not limited to the knowledge of stimulus category or identity. A number of top-down influences can modify perception, such as the perceptual task, attention, expectation, and memory (for a review see [6]). Indeed neuroscientific studies have revealed substantial neural pathways descending from higher-order cortical regions down to sensory processing areas including as early as primary visual cortex and lateral geniculate nucleus. For example, the primary visual cortex receives descending projections from a number of brain areas including the parahippocampal regions, and amygdala [11]. Neurons in the primary visual cortex respond differentially to the same visual stimulus depending on the perceptual task, and stimulus context [7].

While the existence of substantial feedback projections in the visual pathway is well-established and the effect of expectation and perceptual task on visual perception has been well-demonstrated, no research has yet explored the effects of processing numbers on visual perception.

Building on the ESPN survey, we examined the possibility that the cognition of numbers can have an effect on how associated visual stimuli are perceived. Specifically, we investigated the influence of jersey numbers on the perception of athletes’ proportions (slenderness). We examined whether the size of an athlete was perceived differently depending on whether they wear a high or low jersey number.

## Experiment 1

### 1.1 Materials and methods

#### 1.1.1. Participants

A power analysis using the effect size and variance estimates obtained from pilot data indicated a sample size of 35 for 80% power. Experimental procedures were reviewed and approved by the UCLA Institutional Review Board. Participants were recruited from the UCLA Psychology Department Subject Pool and gave informed consent to participate in the experiment. 37 participants completed the experiment. All participants were fluent in English and were asked to not consume any drugs (including alcohol and caffeine) 12 hours prior to the experiment. Participants had normal or corrected to normal vision, and no history of neurological disorders.

#### 1.1.2. Design

A within-subject design was used to compare the ratings of the same football player in two different conditions: low jersey number (10–19), and high jersey number (80–89). A large number of variations in the appearance of the football players was generated by varying the jersey color, skin color, and aspect ratio of players. Examples (different players) and conditions (low number vs. high number) were interleaved so that the observers do not notice that each player was presented twice. Sixty trials in each condition, totaling 120 trials, were presented to each participant.

#### 1.1.3. Stimuli

**Fig 2:**
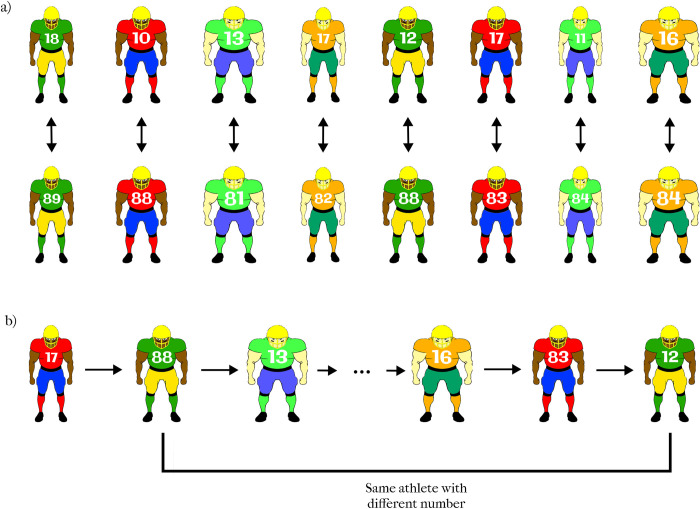
Examples of stimuli used in Experiment 1. a) The same player was presented twice, once with a low number (10–19) and once with a high number (80–89) over the course of the experiment. The difference between the ratings of the two versions of the same player was the dependent variable. b) The athletes varying in aspect ratio, skin color, jersey color, and jersey number were presented in a pseudorandom order across trials.

The stimuli consisted of computer-generated pictures of the frontal view of football players (see Fig 2). The pictures varied in clothing colors, skin tone, and proportion. Clothing color could take on one of four possible color combinations: green/yellow; red/blue; green/blue; or orange/green. Skin tone could take on one of two possible tones: black or white. The proportion could take on one of 10 possible aspect ratios. In order to create different proportions, the width of the players was drawn from a uniform distribution which was defined by a half-open interval (0.35,0.45). Some examples of the stimuli are shown in Fig 2A. Each specific player (with a given aspect ratio, jersey color, skin tone) appeared once with a low number ranging between 10 and 19 (top row of Fig 2A) and another time with a high number ranging between 80–89 (bottom row of Fig 2A). The specific number assigned to each player for each condition was randomly selected from the range 10–19 for the low-number condition and from the range 80–89 for the high-number condition. The font size of the jersey number was adjusted according to the size of the athlete such that the number would not overlap with the body contour, i.e., for smaller athletes, a smaller font size was used (see Fig 2). The resulting 120 trials were presented in a pseudorandom order (see Fig 2B) to reduce bias from extraneous variables. The two versions of the same athlete were never presented one after another to prevent participants from noticing that the same player could appear twice (with different jersey numbers).

#### 1.1.4. Experimental procedure

Due to the pandemic lockdowns, the experiment was delivered online using Pavlovia (https://pavlovia.org/). Subjects were required to complete the experiment in a quiet room without any distractions. The experimenter monitored the participant during the entire experiment using the Zoom software platform (https://zoom.us/). The participants shared their screens with the experimenter, and the experimenter could also see the participant’s face. This way the experimenter could ensure that the participant was not engaged in any other activity on or off the screen and was not distracted during the experiment, and followed instructions throughout the experiment. Participants were instructed to use a laptop or desktop computer and sit in a dim room, reboot their computer prior to the start of the experiment, and not have any other tab open in their browser other than that of the experiment. They were instructed to sit at a comfortable distance from the computer and not change their position during the experiment. The experiment session was followed by a brief questionnaire asking participants about their compliance with the instructions. The experiment took approximately 15 minutes to complete.

**Fig 3:**
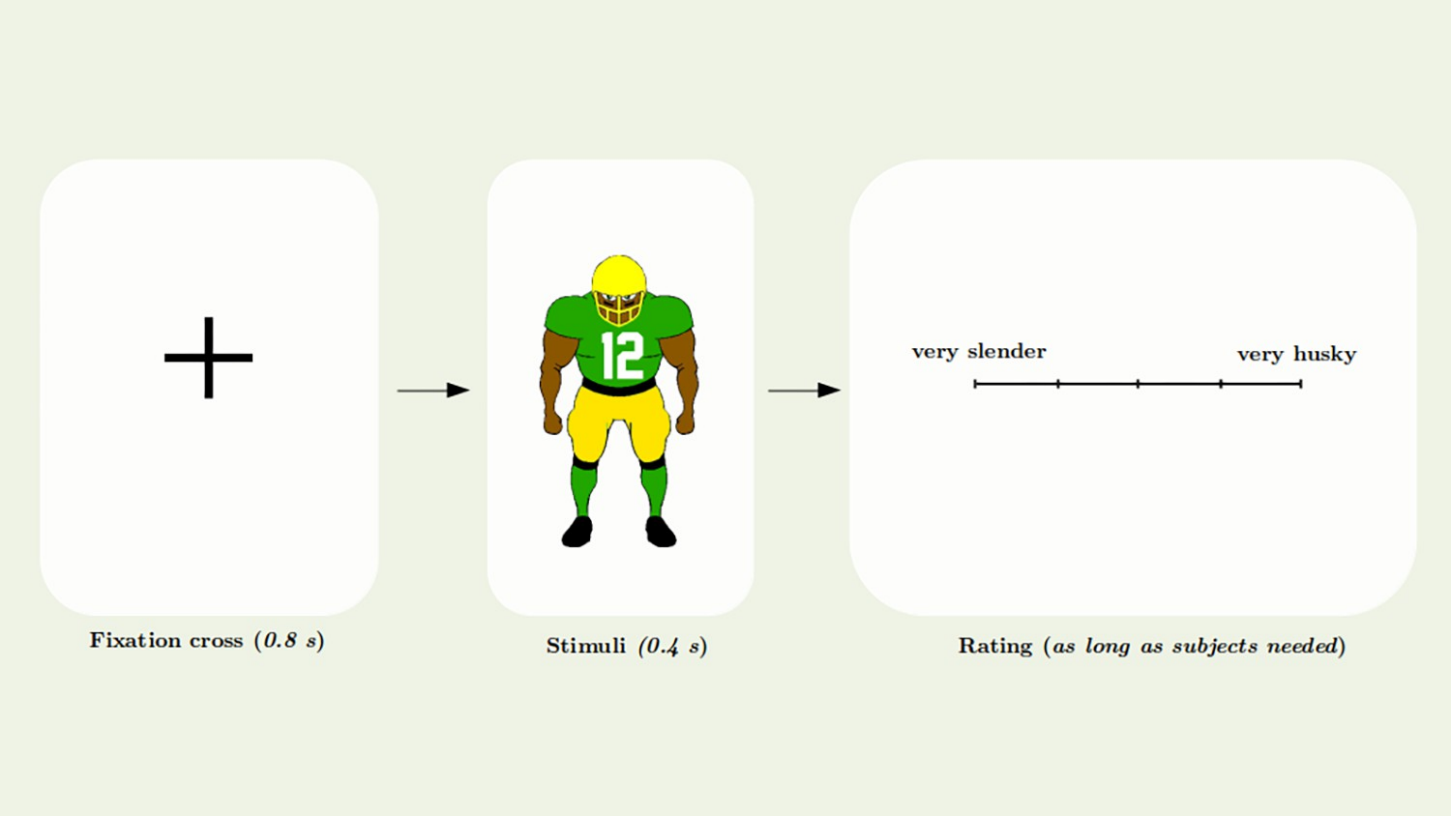
Schematic depiction of a trial. The study participants were first presented with a fixation cross to ensure consistency of gaze across trials. Subsequently, a picture of a football player was briefly displayed. The subjects then had to indicate on a continuous scale how slender or husky they perceived the athlete.

Each trial started with a fixation point which consisted of a plus sign and was presented for 800 ms. This was followed by a football player presented in the center of the screen for 400 ms. The fixation cross was placed at the same position on the screen as the jersey numbers ensuring that the subject’s gaze was located on the number. Subsequently, a scale was presented, also in the center of the screen, for recording the response. Participants were instructed to rate the slenderness of athletes on a continuous scale using their mouse or trackpad. They could choose any value in between the anchor points "very slender" and "very husky". There were no numbers displayed on the response scale. Fig 3 illustrates the procedure of a trial in the experiment.

#### 1.1.5. Data analysis

To analyze the data, the ordinal ratings of participants were converted into numeric scales (0 = very slender, 100 = very husky). Data analysis was carried out in Python (Van Rossum & Drake, 1995) [12] using the SciPy library. For each participant, difference scores were calculated by subtracting the rating of each player with a low number from that of the same player with a high number. The mean of these difference scores was then calculated for each participant. A two-tailed t-test was performed comparing the mean of distribution with zero (df = 36).

### 1.2 Results

**Fig 4:**
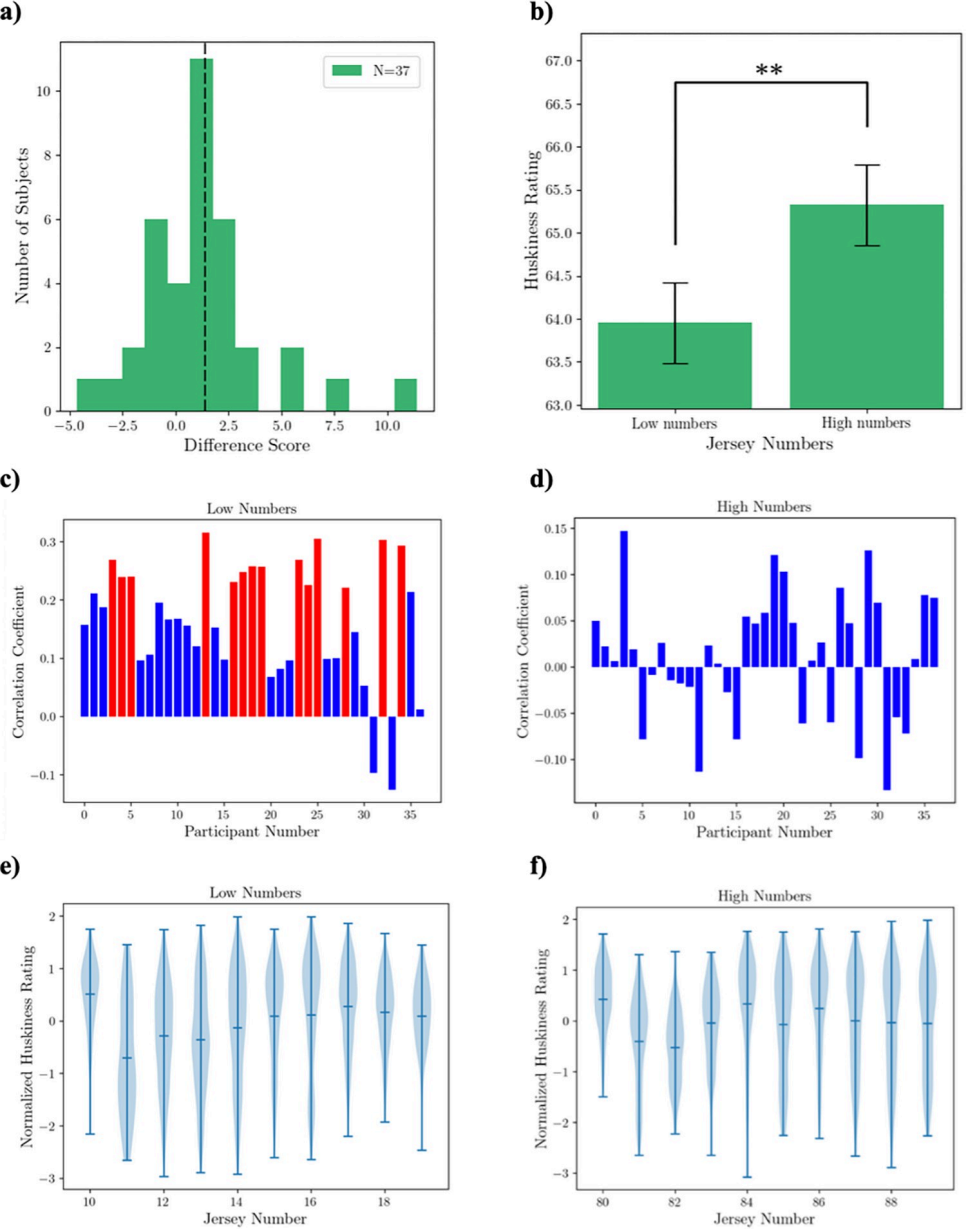
Results of Experiment 1. a) The distribution of mean difference scores across participants. The dashed black line represents the mean difference score of 1.37, which is significantly above zero (p < 0.01). b) Participants’ mean rating scores in the two conditions. The ordinate represents the average ratings of the perceived size of football players (0 = very slender; 100 = very husky) (** denotes p < 0.01). c, d) Pearson’s correlation coefficient r between huskiness ratings and corresponding jersey numbers in the low-number condition (c) and high-number condition (d) across participants. The statistically significant (p<0.05) correlations are shown in red. e, f) Huskiness ratings (normalized within subject) and pooled over all participants as a function of corresponding jersey numbers, for the low-number condition (e) and high-number condition (f).

Fig 4A shows the distribution of mean difference scores (high number—low number) across participants. The mean of this distribution is significantly above zero (M = 1.37; t = 2.90, p = 0.006; Cohen’s d = 0.173), indicating that a high jersey number leads to an increased perceived huskiness. Fig 4B shows the mean rating scores of participants for the perceived huskiness of athletes for the low jersey number condition and high jersey number condition separately.

To probe the relationship between perceived huskiness and jersey number more closely, we examined the correlation between the ratings and the exact jersey numbers within each condition. Fig 4C shows the Pearson’s correlation between raw huskiness ratings and the corresponding jersey numbers in the low number condition for each participant. Fourteen participants had a significant (p<0.05) correlation, shown in red, with a mean correlation coefficient of r = 0.26. The results of the correlations for the high-number condition are shown in Fig 4D. Here, none of the subjects showed a significant correlation. To increase the experimental power for the correlation analyses, we combined the data across all participants. Because different participants may use different ranges of ratings, we normalized (using z transformation) the raw ratings for each participant before pooling the data across subjects. We then computed the Pearson’s correlation coefficient between the normalized ratings and corresponding jersey numbers for each of the two conditions. In the low number condition (Fig 4E), the ratings correlated with the jersey numbers significantly (r = 0.16, p = 0.000). In contrast, in the high number condition, there was no significant correlation between the ratings and jersey numbers (p>0.05). We suspect the ratios of the numbers within each condition may have played a role in the effect size. While the linear range of the jersey numbers was the same (19–10 = 9; 89–80 = 9), the ratio of the largest to smallest number in the high-number condition was small (89:80 ≈ 1.1) as compared to the larger ratio in the low-number condition (19:10 = 1.9), which may have reduced the effect size.

## Experiment 2

The results of Experiment 1 indicate that jersey numbers do influence the perception of an athlete’s huskiness. Athletes with jersey numbers in the 80s were perceived to be more husky (or less slender) than those with jersey numbers in the 10s. While this suggests that the larger magnitude of the number influences the larger size perception, there is an alternative explanation for this effect. Numbers in the 80s all contain the digit 8, whereas numbers in the 10s all contain the digit 1. Digit 8 is wider than digit 1, and therefore on average numbers in the 80s tend to be wider in size than numbers in the 10s. This difference in the visual size of the jersey numbers in the two conditions served as a confounding factor in Experiment 1. In experiment 2, we addressed this issue by controlling for the visual size of the jersey numbers in the two conditions. By choosing numbers that were identical in visual size (for example, 18 vs. 81) and only differed in the numerical value, we removed the visual size confound.

Experiment 1 was performed during the COVID-19 pandemic, when we were not able to collect data from participants in person, due to the closure of campus and the safety restrictions. While we did our best to ensure uniformity in the experimental settings and conditions throughout the experiment and across participants, the degree of influence of uncontrolled parameters on the data was somewhat unclear. Therefore, we aimed to replicate the findings of Experiment 1 by collecting data in the lab under highly controlled conditions, as soon as we became able to collect data from participants in person.

### 2.1 Materials and methods

#### 2.1.1. Participants

A power analysis using the effect size and variance estimates obtained from preliminary data indicated a sample size of 138 for 80% power. The required sample size was larger in this experiment due to the smaller effect size and larger variability. Because the task was tedious, to avoid fatigue and unreliable data, we opted for a relatively small number of trials per subject, and a relatively large number of subjects to obtain the necessary experimental power. Participants were recruited from the UCLA Psychology Department Subject Pool and gave informed consent to participate in the experiment. 149 participants (132 females and 15 males) of the age range 16–37 years (mean 20.10) completed the experiment. Experimental procedures were reviewed and approved by UCLA Institutional Review Board. All participants were instructed not to consume alcohol or drugs 12 hours prior to the experiment. Participants had normal or corrected to normal vision, and no history of neurological disorders.

Two participants were excluded during data analysis, one due to reporting (in a post-session questionnaire) that they had not complied with experimental instructions, and one because of technical problems during the experiment.

#### 2.1.2. Design & stimuli

**Fig 5:**
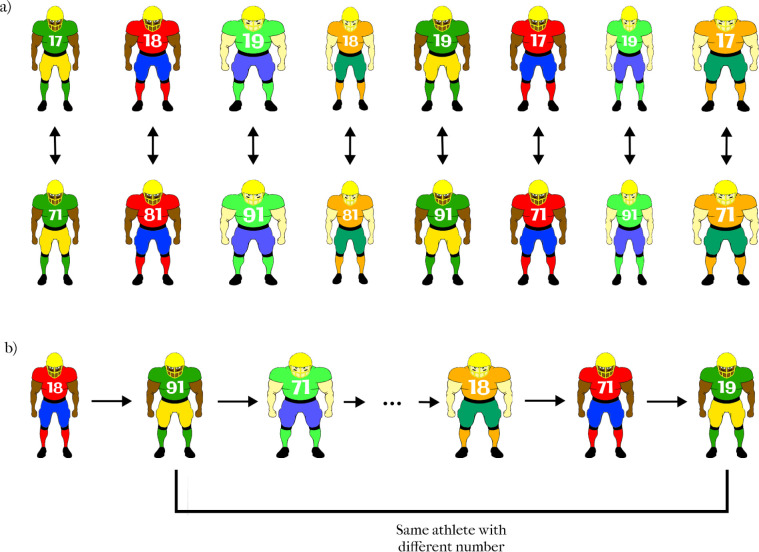
Examples of stimuli used in Experiment 2. a) Over the course of the experiment, each player was presented twice, once with a low number (17, 18, or 19) and once with a large number which was the reverse of the low number (71, 81, or 91). The difference between the ratings of the two versions of each player was the dependent variable. b) The athletes varying in aspect ratio, skin color, jersey color, and jersey number were presented in a pseudorandom order across trials.

The design of the experiment was identical to that of Experiment 1. The stimuli were also identical except for the selection of jersey numbers. Because we wanted to control for the visual size of the numbers, while having one number with a small magnitude (in the teens) and one number with a large magnitude; the low jersey numbers were selected from the set {17, 18, 19} and the high jersey numbers were selected from the set {71, 81, 91}. Each athlete was presented with either {17 and 71} or {18 and 81} or {19 and 91} jersey numbers (see Fig 5A). In this way, the low and high numbers always consisted of the same digits but in the opposite order.

#### 2.1.3. Experimental procedure

The procedure was the same as that of Experiment 1 except for the following. The experiment was conducted in a test room in the laboratory. Participants were seated in front of the screen in a dimly lit room. Stimuli were displayed on a calibrated CRT monitor (85 Hz refresh rate, 800 x 600 resolution) viewed from a distance of 60 cm. Participants were asked to follow the instructions given on the screen. As in Experiment 1, participants were monitored via Zoom throughout the experiment.

### 2.2 Results

**Fig 6:**
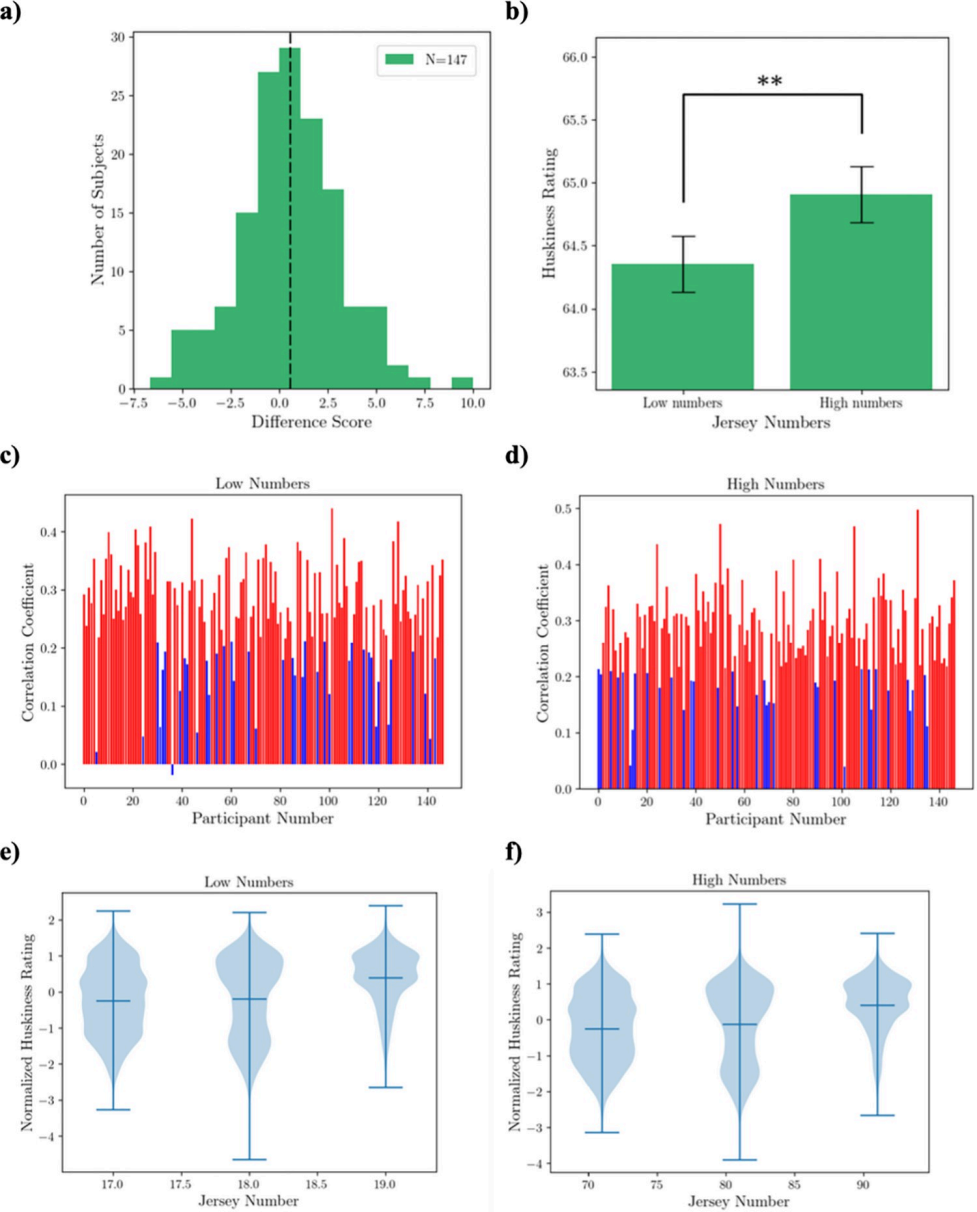
Results of Experiment 2. a) the distribution of mean difference scores pooled over all participants. The dashed black line represents the mean difference score of 0.55, which is significantly above zero (p < 0.01). b) Participants’ mean rating scores in the two conditions. The ordinate represents the average ratings of the perceived size of football players (0 = very slender; 100 = very husky) (** denotes p < 0.01). c, d) Pearson’s correlation coefficient r between huskiness ratings and corresponding jersey numbers in the low-number condition (c) and high-number condition (d) across participants. The statistically significant (p<0.05) correlations are shown in red. e, f) Huskiness ratings (normalized within subject) and pooled over all participants as a function of corresponding jersey numbers, for the low-number condition (e) and high-number condition (f).

Fig 6A shows the distribution of mean difference scores (high number—low number) across participants. The mean of this distribution (M = 0.55) was significantly above zero (t = 2.47; p<0.008; Cohen’s d = 0.067) indicating that large jersey numbers lead to the perception of higher huskiness. Fig 6B shows the mean rating scores of participants for the perceived huskiness of the players for the low jersey number condition and high jersey number condition separately.

Fig 6C shows the Pearson’s correlation between raw huskiness ratings and the corresponding jersey numbers in the low number condition for each participant. As can be seen in the red bars, 107 participants (or 73% of participants) had a significant (p<0.05) correlation, with a mean correlation coefficient of r = 0.30. In the high-number condition (Fig 6D) 111 subjects (75% of participants) showed a significant correlation with a mean correlation coefficient r = 0.30. Fig 6E and 6F show the normalized huskiness ratings as a function of jersey numbers for data pooled across participants in the low number condition and high number condition, respectively. The ratings correlated with the jersey numbers significantly in both the low-number condition (r = 0.25, p = 0.000) and the high-number condition (r = 0.27, p = 0.000).

## Discussion

Observers perceived athletes with low jersey numbers as more slender compared to athletes with high numbers. Experiment 2, which was performed in the lab, replicated the findings of Experiment 1 which was performed online.

In experiment 1, the low number and high number assigned to a given athlete were not matched in visual size. The numbers were selected randomly from a set of low numbers (10–19) and a set of high numbers (80–89). This allowed, for example, a given athlete (with a fixed aspect ratio, skin tone, and jersey color) to possibly take on number 11 in the low number condition, and number 88 in the high number condition. Numbers 11 and 88 are different not only in the numerical magnitude (a cognitive attribute) but also in visual size (a perceptual attribute). One could conceive that the wider numbers (such as 88) would extend in width closer to the body contour compared to narrower numbers (such as 11), and this may have an effect on the perceived size of the athlete. Therefore, the results of Experiment 1 could not tease apart the effect of cognitive representation of magnitude and perceptual representation of the numbers. To address this issue, in Experiment 2, the selection of jersey numbers was restricted to a smaller set of pairs of numbers; wherein the low and high numbers within each pair were composed of the same exact digits and were identical in perceptual size and only differed in cognitive magnitude, for example, 18 and 81.

The findings of Experiment 2 were qualitatively the same as those of Experiment 1, confirming that the cognitive factor of jersey number magnitude does influence the perceived slenderness of athletes. However, the effect size in Experiment 2 was smaller than that of Experiment 1, suggesting that the visual width of the numbers may have also contributed to the effect observed in Experiment 1.

Examination of the linear correlation between subjective ratings of huskiness and the exact Jersey number within each condition revealed a significant correlation in the low-number condition in Experiment 1 and a significant correlation in both conditions in Experiment 2 (r = 0.25 for low numbers and r = 0.27 for high numbers, p = 0.000). This strong and highly robust correlation between the ratings and jersey numbers, despite the small range of numbers used in the experiment, confirms the role of jersey numbers in the perception of size. The absence of a correlation in the high-number condition in Experiment 1 is curious and requires further investigation. Future studies using a larger set of jersey numbers (see below) can shed light on possible non-linearities in the association.

**Fig 7:**
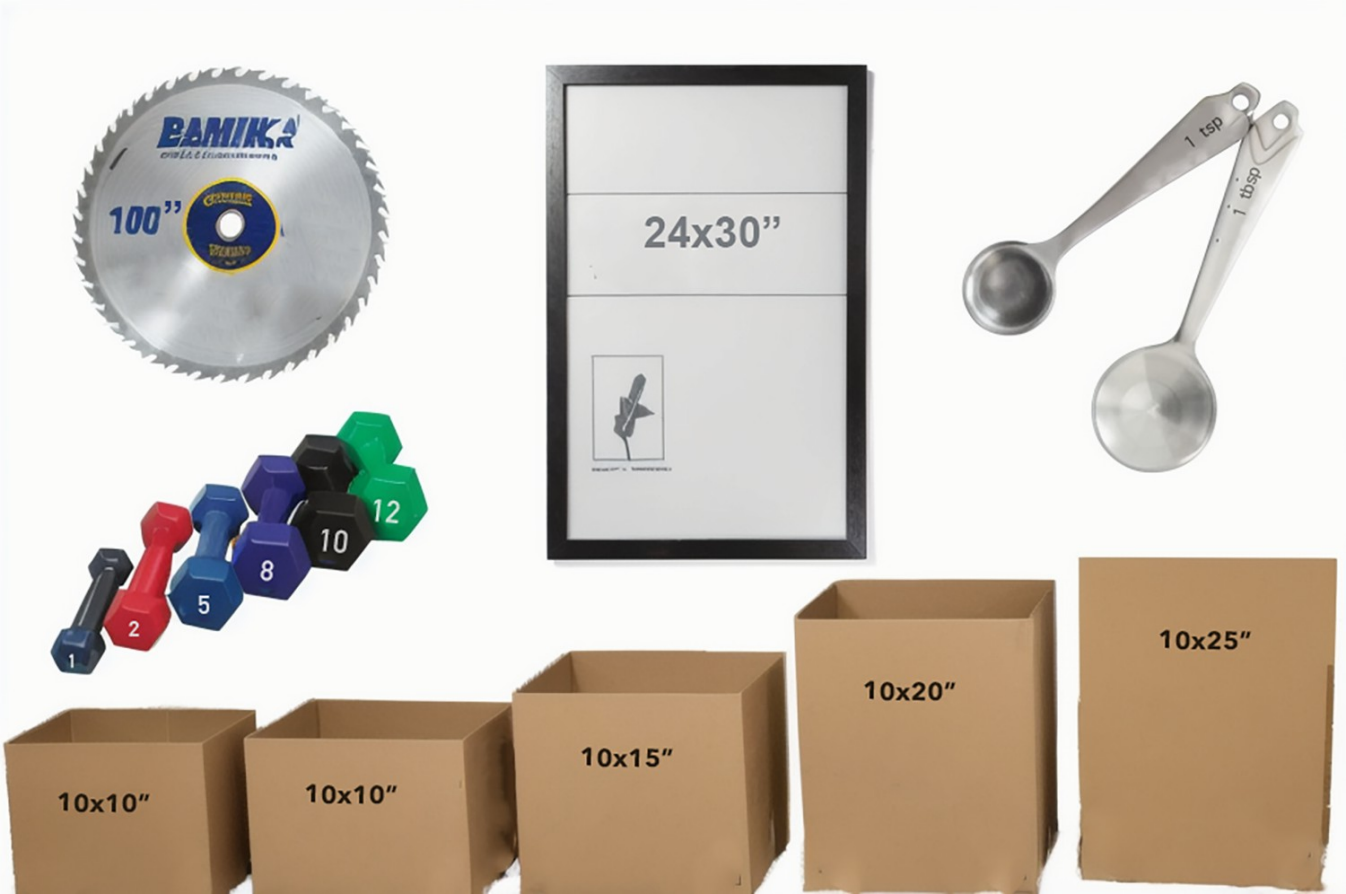
Examples of objects in which a number written on the object represents the size of the object. In the case of the dumbbells, the number represents the mass but the mass frequently is correlated also with size.

Altogether, the results of the two experiments show that low jersey numbers make the athletes appear more slender. We conjecture that this effect results from learned associations between numbers and size attributes of objects in daily life (Fig 7). The assumption that statistical regularities of numbers and size attributes are stored in the brain is consistent with previous literature demonstrating statistical learning being a fundamental and ubiquitous learning mechanism (see e.g. [13–15]).

Learned over long timescales, the joint probabilities of numbers and size attributes (see Fig 5) may be turned into prior expectations in the brain, and thus modulate inference of perceptual properties such as size. This assumption links to Bayesian inference models, in which perception is shaped by current evidence, or sensory input, as well as prior knowledge.

The existence of top-down influences of cognition on perception has recently been challenged by holding pitfalls accountable for reported findings [16]. The current study, however, demonstrates a surprising example of cognitive penetration and supports, together with a wealth of previous evidence (see [6, 8] for overviews), the notion of top-down influences on perception. In particular, the current finding reveals that the cognition of numbers can quantitatively alter body size perception.

The data collected in both online and laboratory settings produced similar results. These findings are in line with studies showing no significant differences in the results of online and in-lab experiments [17]. Earlier studies have established that the perceptual nature of the experiment does not get significantly influenced by the experimental setting [18], and our results further support these findings.

Future research should investigate the generality of the reported effect by investigating the influence of written numbers on the size perception of other objects and examining the influence of written numbers in other perceptual domains. The conjecture about the statistical learning of joint probabilities of numbers and size can be tested by exposing observers to opposite statistical regularities (e.g., large numbers written on small objects, and vice versa) and examining if the effect becomes weaker or reverses after extended exposure (similar to modifying the ‘light-from-above’ prior [19]).

In this study, we compared two distinct sets of numbers, one in the 80s and one in the teens. This choice was inspired by the real-life jersey numbers used in real-life football teams and the ESPN survey about the numbers. But this choice also increased the effect size and experimental power. To examine the relationship between jersey numbers of body size perception in more depth, future studies should include a more continuous set of numbers, for example, ranging from 1 to 100, and examine the quantitative nature of the correlation between ratings of huskiness and jersey number. Finally, future studies may examine the effect of the directionality of the response scale by presenting “very husky” on the left and “very slender” on the right end of the scale.
